# A longitudinal study on the COVID-19 pandemic and its divergent effects on social participation and mental health across different study groups with and without mental disorders

**DOI:** 10.1007/s00127-021-02025-9

**Published:** 2021-02-10

**Authors:** Eduard Mergel, Matthias Schützwohl

**Affiliations:** Department of Psychiatry and Psychotherapy, Technische Universität Dresden, Fetscherstraße 74, 01307 Dresden, Germany

**Keywords:** COVID-19, Coronavirus, Social participation, Psychological well-being, Mental health, Mental disorder

## Abstract

**Purpose:**

With the rapid spread of COVID-19 and the restrictions imposed in many parts of the world, there is growing concern about its impact on mental health. This longitudinal study investigated the social participation, social inclusion and psychological well-being in adults with and without mental disorders before the beginning of the pandemic, as well as during and after strict lockdown restrictions in Germany.

**Methods:**

The sample (*n* = 106) consisted of three groups: participants with a chronic mental disorder, with an acute mental disorder, and without a mental disorder at the time of the initial survey. Parameters of interest were assessed using the Measure of Participation and Social Inclusion for Use in People with a Chronic Mental Disorder (F-INK), the Index for the Assessment of Health Impairments (IMET) and the Brief Symptom Inventory (BSI-18).

**Results:**

The perceived impairments in social participation and the associated changes in behaviour varied depending on the presence of a mental disorder at the time of the initial survey and were largely temporary, i.e. limited to the period of strict lockdown restrictions. We found no further detrimental effects on mental health 4 weeks after lockdown or later, when a policy of physical distancing was in place.

**Conclusion:**

Overall, our findings suggest a general resilience to the official restrictions and measures as well as the pandemic itself. However, further efforts are needed to improve the situation of people with chronic mental disorders and their limited opportunities for social participation.

**Supplementary Information:**

The online version contains supplementary material available at 10.1007/s00127-021-02025-9.

## Introduction

The global spread of COVID-19 and the various measures taken in response, such as lockdown restrictions or physical distancing policies, have raised concerns about its impact on mental health. Since the beginning of the pandemic, the number of studies dealing with the psychological consequences is growing rapidly.

With respect to the general population, some studies have found a negative impact on mental health outcomes. For example, in a cross-sectional study carried out in Italy during the first three weeks after the begin of lockdown restrictions, it was found that the duration of lockdown restrictions as well as the quality of domestic living situations had a negative impact on the well-being of residents. Social contacts, both online and offline, were found to mitigate this effect [1]. The results of a cross-sectional study conducted in China further showed that those individuals who were unable to pursue any professional activity were more likely to experience psychological distress four weeks after the begin of lockdown restrictions [2]. Another study from the UK found significant increases in mental health problems in April 2020 compared to 2017–2019 [3]. Finally, findings from a study conducted in Spain indicated a detrimental effect of the pandemic during its first weeks [4].

In contrast, other studies found a predominant resilience in the general population. A study from New Zealand, for example, compared the well-being of propensity score matched samples from population surveys before and after a nationwide lockdown. It was found that individuals surveyed after the lockdown reported only slightly increased specific psychological distress but were also observed to possess a high degree of resilience [5]. Likewise, data from a panel in Germany showed unchanged levels of well-being in April 2020 compared to previous surveys completed between 2016 and 2019 [6]. Similar results suggesting resilience were found in a survey examining the mental and social health in the older German population [7], as well as a sample of the general population in Dresden before and four weeks after lockdown restrictions [8].

A recent systematic review revealed that knowledge regarding the impact of COVID-19 on patients with pre-existing psychiatric disorders remains scarce [9]. Here too, the findings of the few existing studies are mixed. Results of a study from China showed that, compared to psychiatric patients, control subjects experienced less psychological stress during the early weeks of the pandemic while lockdown restrictions were in place. However, the authors of this cross-sectional study were unable to prove that these findings were a direct result of the pandemic and/or lockdown restrictions [10]. According to the results of another cross-sectional study involving several countries, more than half of the participants who reported a previous mental disorder also stated that their condition had worsened due to the COVID-19 pandemic [11]. In contrast, in our own study conducted in Germany, we found no detrimental effects on the psychological well-being of mentally ill participants four weeks after the implementation of lockdown restrictions, compared to their base levels recorded in surveys before the lockdown [8]. These results were consistent with the observations made by Martinelli and Ruggeri [12] in Italy.

In part, these conflicting findings may be attributed to the fact that studies differed in respect to numerous factors, such as the study design, the instruments used, or the way subjects were recruited. However, another explanation could also be the numerous factors that differed between the countries at the time the studies were conducted, i.e. the extent to which the population was affected by the pandemic including the number of infections or related deaths, the severity of the measures taken by the government or the capacity of the health care system. For example, in a multinational study conducted at a relatively early stage of the pandemic, participants who were dissatisfied with the measures of their government reported higher worries and fears that were in turn related to experiencing more distress [13]. Similarly, trust in governmental actions to tackle COVID-19 was negatively associated with psychological distress as well as generalised anxiety and depression symptoms in a large German sample [14]. A nationwide study in China further found that the self-reported level of distress was highest in the region including Hubei which was most severely affected by the spread of COVID-19 compared to other regions. Other factors affecting the level of distress included the local efficiency of the public health care system and the availability of medical resources [15].

The above-mentioned factors differ not only across countries but may also change over time. We therefore conducted a second follow-up survey to our initial study to investigate the course of the effects on various clinical and social psychiatric variables in participants with and without acute or chronic mental disorders. The aim of the present study was to assess the same variables within the same individuals before the beginning of the pandemic as well as during and after strict lockdown restrictions to describe the effects of COVID-19 for each group individually.

## Materials and methods

### Materials

The primary instrument used in this study was the Measure of Participation and Social Inclusion for Use in People with a Chronic Mental Disorder (F-INK) [16]. The questionnaire has a modular structure and allows for the recording of socio-demographic and clinical variables. Through the “participation module”, the questionnaire is able to measure the behaviour-related extent of social participation, operationalised as the frequency with which 32 individual activities (e.g. watching television, meeting friends) were carried out. Answers are coded 4 = almost every day, 3 = multiple days per week, 2 = approx. once per week, 1 = approx. once or twice per month and 0 = never in the last four weeks, which can then be compounded into a total score. The “inclusion module” uses self-assessment on a 4-point Likert scale to record feelings of belonging (0 = not at all; 1 = little; 2 = moderately; 3 = very much) as it is experienced in different social contexts (e.g. in the home living environment or with immediate family members). These self-assessment scores can also be summarised to a total value.

Despite its name, the Index for the Assessment of Health Impairments (IMET) [17] assesses the extent of perceived impairments in social participation (e.g. everyday activities like family/household obligations, recreational and leisure activities) during the assessment period using an 11-point scale (0 = no impairment; 10 = no activity possible anymore). Assessment is completed regardless of whether the impairments are due to personal reasons or as a direct consequence of lockdown restrictions.

The Brief Symptom Inventory (BSI-18) [18] is a self-assessment tool which measures depression, anxiety and somatisation, with six items each. A 5-point Likert scale (0 = not at all; 4 = extremely) is used to assess the extent of participants’ subjective stress experiences. In contrast to the other instruments, these items refer to the seven days prior to the survey.

In addition to the aforementioned questionnaires, participants were asked at follow-up to answer specific questions regarding their subjective concerns, as well as the subjective possibilities of events in relation to the coronavirus pandemic (e.g. likelihood of contracting coronavirus). These questions were taken from the “COVID-19 Snapshot Monitoring (COSMO)” survey [19].

### Participants

As part of an ongoing research study aiming to apply the F-INK to comprehensive statistical test studies, participants had been recruited since August 2019. Participants were assigned to one of three groups: Group 1 consisted of individuals with a chronic mental disorder. To be assigned to this group, participants must not have been acutely ill in the four weeks prior to the initial survey. These participants were accessed via institutions providing assisted living facilities. Group 2 consisted of individuals who were receiving acute psychiatric or psychotherapeutic treatment at the time of the initial survey. These participants were recruited from consecutive acute admissions to the Department of Psychiatry and Psychotherapy at the TU Dresden. The initial survey was conducted within three days of admission. Group 3 consisted of members of the general public, who at the time of the initial survey presented with neither acute nor chronic mental disorders. These participants were recruited from the Dresden general population with the aim of being as representative as possible in a complex recruitment process using cluster and stratification processes. For all three groups, inclusion criteria were an age between 18 and 65 years, as well as the ability to provide an informed declaration of consent.

A total of *n* = 174 individuals that had previously been interviewed between August 2019 and early March 2020 as part of the initial study were invited to participate in the first follow-up survey on April 17, 2020. Participants were instructed to provide answers based on the four weeks following the start of strict lockdown restrictions (March 23, 2020–April 20, 2020), unless stated differently in a specific item or questionnaire. Responses received between April 22, 2020 and May 13, 2020 were included in the evaluations. A total of *n* = 132 participants completed the initial and the first follow-up survey, with response rates being 72.0% for Group 1, 65.2% for Group 2 and 91.4% for Group 3.

Three months later (July 17, 2020), the *n* = 132 respondents were invited to take part in a second follow-up survey. Again, participants were instructed to relate their answers to the last four weeks (June 22, 2020–July 19, 2020), unless stated differently in a specific item or questionnaire. A total of *n* = 106 participants completed the second follow-up and thus all three surveys, with response rates being 75.0% for Group 1, 69.8% for Group 2 and 92.5% for Group 3. Systematic comparisons of the basic socio-demographic characteristics between the drop-outs and the completers of all three surveys revealed significant differences in Group 2 only: here, the drop-outs were significantly younger (*M* = 35.8, SD = 12.4) than the completers (*M* = 43.4, SD = 11.9) [*t*(64) = -2.53, *p* = 0.014]. The average response period between the initial and the first (and second) follow-up survey was 18.3 (30.8) weeks and ranged from 4 to 36 (17–48) weeks, whereby the three groups did not differ in mean periods.

### COVID-19 cases and pandemic decrees in the study region

In the period to which the study participants should refer their answers at the first follow-up survey, the number of identified COVID-19 cases in Dresden rose from 153 on March 23, 2020 to 523 on April 20, 2020, corresponding to 370 new infections in four weeks [20]. With the aim of curbing the spread of SARS-CoV-2 infections, several lockdown restrictions were put in place in Dresden from March 22, 2020. Leaving one’s own home was only permitted for valid reasons, such as professional employment or for “obtaining necessary household supplies”. It was advised that contact with persons outside of one’s personal household should be kept “to the bare minimum”, while gatherings and celebrations with multiple individuals were prohibited. Sport and exercise in the fresh air were permitted “alone or with members of the same household” [21]. These lockdown restrictions were eased on April 17, 2020 and were replaced by social distancing policies, including an outright ban on group gatherings. Individuals were permitted to leave their households at free will, but only “in the accompaniment of partner(s) or family members of the same household” [22].

In the four weeks prior to the second follow-up survey, the number of identified cases in Dresden increased only slightly from 624 on June 22, 2020, to 631 on July 19, 2020, corresponding to 7 new infections [20]. During this period, most restrictions that applied during the first follow-up had already been largely relaxed. Private gatherings were allowed with members of another household or up to ten other people when in public, but without number limits when at home. With a few exceptions, there was an extensive opening of service providers and other enterprises, institutions, sports facilities, food services, hotels, businesses and shops, as well as public transports and certain events (under observation of hygiene rules). Nevertheless, people were generally encouraged to limit unnecessary physical contact, to keep at least 1.5 m away from others whenever possible, and to follow hygiene recommendations. Wearing a mouth and nose covering was only mandatory when using public transport or visiting shops [23].

### Statistical analyses

Sampling characteristics were reported descriptively. Due to the large differences between the groups defined by the inclusion criteria, only within-group variations were of interest. Therefore, paired samples *t* tests and repeated measures ANOVAs with post hoc pairwise comparisons were conducted for each group separately to compare the results from the initial and both follow-up surveys. In case the assumption of sphericity was violated, a Huynh–Feldt correction was applied. An alpha level of 0.05 was used to assess for statistical significance, while the Bonferroni–Holm correction was used to adjust the α-error, which is less conservative than the Bonferroni correction. These adjustments were made for each of the analyses of the questionnaires. Due to the small number of participants per group, we additionally reported the *f* values in Tables 3, 4, and the Supplementary Tables as a measure of the experimental effect. We used only complete cases with data on all three survey dates for our analyses, as the different response rates between the groups suggested that drop-outs over the course of the study were not random. The analyses were conducted using IBM SPSS Statistics 27.

## Results

### Participant demographics

Table 1 shows the socio-demographic and clinical characteristics of the study participants. As expected, participants with a chronic mental disorder (Group 1) differed from participants in Groups 2 and 3 in several aspects. Study participants in Group 3 (i.e. those with neither chronic nor acute mental disorders at the time of the initial survey) were predominantly female and were seemingly more affected by the COVID-19 restrictions in their professional lives, compared to participants in Groups 1 and 2.

**Table 1:** Study participants

Variable	Gr. 1 (*n* = 25–27)		Gr. 2 (*n* = 29–30)		Gr. 3 (*n* = 46–49)	
Gender (% female)	13 (48.1)		18 (60.0)		37 (75.5)	
Age (in years) M (SD)	49.7 (13.1)		44.0 (11.8)		41.0 (13.9)	
Marital status (% single)	16 (64.0)		18 (60.0)		23 (46.9)	
Partner relationship (% alone)	21 (80.8)		14 (46.7)		17 (34.7)	
Living situation (% living alone)	17 (63.0)		17 (56.7)		13 (26.5)	
If not living alone						
Spouse	1 (3.7)		10 (33.3)		29 (59.2)	
Roommate(s)	8 (29.6)		1 (3.3)		3 (6.1)	
Parent(s)	1 (3.7)		0 (0.0)		4 (8.2)	
Child(ren)	1 (3.7)		7 (23.3)		17 (34.7)	
Other person(s)	0 (0.0)		0 (0.0)		1 (2.0)	
Highest vocational qualification (% none)	6 (22.2)		5 (16.7)		1 (2.0)	
Current employment situation						
Student	1 (3.7)		2 (6.7)		4 (8.2)	
Working	0 (0.0)		14 (46.7)		37 (75.5)	
Sheltered employment	4 (14.8)		2 (6.7)		0 (0.0)	
Unable to work for more than 6 weeks	5 (18.5)		5 (16.7)		0 (0.0)	
Housekeeper	0 (0.0)		0 (0.0)		1 (2.0)	
Retired	17 (63.0)		3 (10.0)		5 (10.2)	
Working on a voluntary basis	2 (7.4)		0 (0.0)		1 (2.0)	
Informally employed	0 (0.0)		0 (0.0)		1 (2.0)	
Unemployed	4 (14.8)		5 (16.7)		3 (6.1)	
Other	1 (3.7)		3 (10.0)		3 (6.1)	
Effects of SARS-CoV-2-related measures on the occupational situation						
No change	23 (88.5)		22 (75.9)		28 (57.1)	
Reduction of working hours	1 (3.8)		2 (6.9)		5 (10.2)	
Increase in working hours	0 (0.0)		0 (0.0)		6 (12.2)	
More working from home	1 (3.8)		1 (3.4)		8 (16.3)	
Released from work without pay	0 (0.0)		0 (0.0)		1 (2.0)	
Released from work with pay	0 (0.0)		1 (3.4)		0 (0.0)	
Dismissed	0 (0.0)		1 (3.4)		1 (2.0)	
Other	1 (3.8)		3 (10.3)		5 (10.2)	
Presence of a mental disorder (in %)	27 (100.0)		30 (100.0)		3 (6.1)	
Classification according to ICD-10						
F2 Schizophrenia et al.	10 (38.5)		1 (3.6)		0 (0.0)	
F3 Affective disorders	11 (42.3)		21 (75.0)		3 (100.0)	
F4 Anxiety disorders et al.	7 (26.9)		11 (39.3)		0 (0.0)	
F6 Personality disorders	5 (19.2)		2 (7.1)		0 (0.0)	
Chronicity of mental disorder						
< 2 years	0 (0.0)		1 (4.0)		0 (0.0)	
2–5 years	0 (0.0)		4 (16.0)		1 (33.3)	
6–10 years	6 (26.1)		6 (24.0)		1 (33.3)	
> 10 years	17 (73.9)		14 (56.0)		1 (33.3)	
In treatment for mental health problems, initial survey (in %)	24 (88.9)		30 (100.0)		0 (0.0)	
In treatment for mental health problems, first follow-up survey (March 23–April 20) (in %)	26 (96.3)		27 (93.1)		1 (2.0)	
In treatment for mental health problems, second follow-up survey (June 22–July 19) (in %)	26 (96.3)		27 (90.0)		0 (0.0)	

### COVID-19 specific questions

At the time of the second follow-up survey, 16 participants (5 from Group 1, 1 from Group 2 and 10 from Group 3) stated knowing people in their immediate social environment who were or had been infected with the SARS-CoV-2. Only one participant from Group 1 reported that she had been infected herself. Across all three groups, the self-estimated probability of getting infected with the SARS-CoV-2, as well as the evaluation of one’s own susceptibility to an infection and the expected severity of its consequences remained medium (Table 2). Most participants indicated that they followed the official measures and recommendations “rather strictly” or “very strictly”, with no significant differences in mean values between the two follow-up surveys (Table 2) or between groups, neither in the first (*F*(2, 103) = 1.21, *p* = 0.302) nor in the second follow-up survey (*F*(2, 103) = 1.40, *p* = 0.252).

**Table 2:** Impact, probability and handling of measures and recommendations. The Bonferroni–Holm method was applied to obtain the adjusted *p* values

	First follow-up survey (March 23–April 20)	Second follow-up survey (June 22–July 19)				
	*M* (SD)	*M* (SD)	*t*	*df*	*p*	*p*_adj_
If you have not been infected yet: what do you consider to be your own probability of getting infected with the coronavirus?^1^						
Gr. 1 (*n* = 25)	2.44 (1.3)	2.56 (1.2)	− 0.49	24	.632	1.000
Gr. 2 (* n* = 30)	2.63 (1.1)	2.40 (0.9)	1.42	29	.165	.496
Gr. 3 (* n* = 49)	2.80 (0.9)	2.35 (0.9)	2.53	48	.015	.059
If you have not been infected yet: from your point of view, how severe would the consequences of an infection with the coronavirus be for you?^2^						
Gr. 1 (* n* = 26)	3.65 (1.3)	3.19 (1.3)	1.76	25	.090	.359
Gr. 2 (* n* = 29)	2.97 (1.3)	2.72 (1.1)	1.02	28	.316	.631
Gr. 3 (* n* = 49)	2.57 (1.0)	2.53 (1.1)	0.40	48	.687	1.000
If you have not been infected yet: how susceptible do you consider yourself to an infection with the coronavirus?^3^						
Gr. 1 (* n* = 26)	2.92 (1.2)	2.96 (1.1)	− 0.18	25	.857	1.000
Gr. 2 (* n* = 30)	2.60 (1.0)	2.67 (1.1)	− 0.47	29	.645	.645
Gr. 3 (* n* = 49)	2.57 (1.0)	2.61 (0.9)	− 0.27	48	.785	1.000
How strictly do you follow the official measures and recommendations to protect yourself from an infection with the coronavirus or to prevent the spread of the coronavirus?^4^						
Gr. 1 (* n* = 27)	4.33 (1.0)	4.11 (1.0)	0.81	26	.425	1.000
Gr. 2 (* n* = 30)	4.00 (0.8)	3.73 (0.8)	1.61	29	.118	.473
Gr. 3 (* n* = 49)	4.08 (0.8)	3.90 (0.8)	1.84	48	.071	.214

^1^[1] = very unlikely–[5] = very likely

^2^[1] = not severe at all–[5] = very severe

^3^[1] = not susceptible at all–[5] = very susceptible

^4^[1] = not strictly at all–[5] = very strictly

### Perceived impairments in social participation (IMET)

Participants with a chronic mental disorder (Group 1) did not report any significant additional impairments in social participation in comparison to the initial survey, neither during the lockdown restrictions prior to the first follow-up survey, nor during the physical distancing policy prior to second follow-up survey (Table 3). Participants who were receiving acute psychiatric or psychotherapeutic treatment at the time of the initial survey (Group 2) experienced significantly less impairment in three out of nine areas during the lockdown restrictions and in additional five areas during the physical distancing policy, compared to the initial survey (Table 3). In contrast, participants in Group 3 indicated that they felt significantly more impaired in five out of nine areas during the lockdown restrictions, compared to the initial survey. While three of the more impaired areas returned to normal during the physical distancing policy, the remaining two areas improved but were still significantly impaired in comparison to the initial survey (Table 3).

**Table 3:** Perceived impairments in social participation (IMET). In case the sphericity assumption was violated, a Huynh–Feldt correction was applied. The Bonferroni–Holm method was applied to obtain the adjusted *p* values. Per row, means followed by a different letter significantly differ at 5% level according to post hoc tests

	Initial survey	First follow-up survey (March 23–April 20)	Second follow-up survey (June 22–July 19)					
	*M* (*SD*)	*M* (*SD*)	*M* (*SD*)	*F*	*df*	*f*	*p*	*p*_adj_
Gr. 1 (* n* = 19–27)								
Usual activities of daily life^1^	2.35 (2.6)	2.23 (2.4)	3.38 (3.0)	2.59	2	0.32	.085	.683
Family and domestic responsibilities^1^	3.70 (2.9)	3.09 (3.0)	4.70 (2.9)	2.57	2	0.34	.088	.683
Getting things done outside of home^1^	2.67 (2.6)	3.17 (3.0)	3.33 (2.9)	0.67	2	0.17	.516	1.000
Daily tasks and obligations^1^	3.50 (3.0)	3.87 (3.4)	4.25 (2.8)	0.62	2	0.16	.540	1.000
Recreation and leisure^1^	3.92 (3.0)	4.42 (3.9)	3.83 (3.1)	0.24	2	0.10	.790	1.000
Social activities^1^	4.13 (3.6)	6.08 (4.1)	4.17 (3.7)	3.65	2	0.40	.034	.304
Close personal relationships^1^	4.48 (3.6)	5.09 (4.0)	4.35 (3.4)	0.45	2	0.14	.642	1.000
Sex life^1^	4.42 (4.5)	5.68 (4.4)	6.26 (4.3)	1.51	2	0.29	.234	1.000
Stress and extraordinary strain^2^	5.95 (3.3)	5.63 (3.3)	6.00 (3.7)	0.14	2	0.09	.870	1.000
Gr. 2 (* n* = 26–30)								
Usual activities of daily life^1^	3.50^a^ (3.0)	1.43^b^ (1.9)	1.53^b^ (1.8)	13.97	1.6	0.69	< .001	.001
Family and domestic responsibilities^1^	4.07^a^ (3.2)	2.41^b^ (2.4)	2.17^b^ (2.1)	7.31	2	0.51	.002	.009
Getting things done outside of home^1^	3.77^a^ (3.1)	2.53^ab^ (2.8)	2.07^b^ (2.1)	5.21	2	0.42	.008	.033
Daily tasks and obligations^1^	4.14^a^ (3.1)	2.79^ab^ (2.9)	2.43^b^ (2.2)	4.93	2	0.43	.011	.033
Recreation and leisure^1^	5.60^a^ (3.4)	4.67^a^ (3.1)	2.97^b^ (2.5)	7.20	2	0.50	.002	.009
Social activities^1^	5.63^a^ (3.6)	6.20^a^ (3.8)	4.03^b^ (3.4)	4.12	2	0.38	.021	.043
Close personal relationships^1^	4.90^a^ (3.3)	5.07^a^ (3.4)	2.47^b^ (2.8)	8.44	2	0.54	.001	.004
Sex life^1^	4.81 (3.9)	4.92 (3.8)	4.38 (3.6)	0.22	2	0.10	.802	.802
Stress and extraordinary strain^2^	7.22^a^ (2.5)	5.30^b^ (2.6)	4.52^b^ (2.6)	12.51	2	0.69	< .001	< .001
Gr. 3 (* n* = 45–49)								
Usual activities of daily life^1^	0.29 (0.8)	0.22 (1.1)	0.16 (0.5)	0.30	1.6	0.08	.693	.693
Family and domestic responsibilities^1^	0.63 (1.5)	0.80 (1.9)	0.31 (0.8)	1.49	1.6	0.18	.233	.466
Getting things done outside of home^1^	0.55^a^ (1.4)	3.14^b^ (3.1)	0.80^a^ (1.3)	30.55	1.4	0.80	< .001	< .001
Daily tasks and obligations^1^	0.49^a^ (1.2)	2.04^b^ (2.7)	0.69^a^ (1.3)	13.10	1.6	0.52	< .001	< .001
Recreation and leisure^1^	0.90^a^ (1.5)	5.25^b^ (3.2)	1.73^c^ (2.2)	52.71	1.8	1.06	< .001	< .001
Social activities^1^	0.84^a^ (1.5)	7.76^b^ (3.3)	2.37^c^ (2.6)	119.65	2	1.58	< .001	< .001
Close personal relationships^1^	0.86^a^ (1.7)	3.31^b^ (3.2)	0.86^a^ (1.6)	21.83	1.7	0.67	< .001	< .001
Sex life^1^	2.09 (2.8)	2.80 (3.5)	1.80 (2.5)	2.19	1.7	0.22	.126	.377
Stress and extraordinary strain^2^	2.35 (2.4)	1.49 (1.5)	1.94 (1.9)	3.38	2	0.27	.038	.153

.10 ≤ *f* < .25 = small effect, .25 ≤ *f* < 0.40 = medium effect, *f* ≥ .40 = large effect

^1^[0] = no impairment–[10] = no activity possible anymore

^2^[0] = can bear the strain–[10] = can no longer bear the strain

### Participation and inclusion (F-INK)

Across the three surveys, the overall level of participation, as measured by the “participation module” of the F-INK, did not change significantly in Groups 1 or 2 (Table 4). Across all survey dates, participants of Group 1 carried out 31 out of 32 solo activities with unchanged frequency. In Group 2, this was the case for 30 out of 32 activities. Participants in Group 1 attended less parties in the four weeks prior to the first and second follow-up surveys, while participants in Group 2 visited bars or restaurants and concerts, cinemas, etc. with reduced frequency (Supplementary Tables 4.1.1 and 4.1.2). In strong contrast, participants in Group 3 reported significant changes in their overall level of participation across the three surveys. A post hoc pairwise comparison showed significant differences between the initial and the first follow-up survey, as well as between the first and the second follow-up surveys (Table 4). Out of the 32 solo activities recorded, 15 were carried out with notably less frequency during the lockdown restrictions compared to the initial survey. The frequency of 13 of those 15 activities returned to normal in the four weeks prior to the second follow-up survey, however, participants in Group 3 were still going for more walks and visiting concerts, cinemas, etc. less frequently (Supplementary Table 4.1.3).

**Table 4:** Participation, inclusion (F-INK) and mental health problems (BSI-18). In case the sphericity assumption was violated, a Huynh–Feldt correction was applied. The Bonferroni–Holm method was applied to obtain the adjusted *p* values. Per row, means followed by a different letter significantly differ at 5% level according to post hoc tests

	Initial survey	First follow-up survey (March 23–April 20)	Second follow-up survey (June 22–July 19)					
	*M* (SD)	*M* (SD)	*M* (SD)	*F*	*df*	*f*	*p*	*p*_adj_
Gr. 1 (*n* = 24–27)								
F-INK								
Scale ‘participation'	1.02 (0.4)	0.92 (0.5)	0.98 (0.4)	0.78	2	0.17	.466	.932
Scale ‘inclusion'	1.86 (0.6)	1.85 (0.7)	1.80 (0.6)	0.33	2	0.11	.723	.932
BSI-18								
Scale ‘somatization'	0.59 (0.5)	0.62 (0.5)	0.71 (0.7)	0.72	2	0.17	.493	1.000
Scale ‘depression'	1.09 (1.0)	1.14 (0.9)	1.02 (0.9)	0.31	2	0.11	.738	1.000
Scale ‘anxiety'	0.90 (0.8)	0.91 (0.8)	0.91 (0.7)	0.01	2	0.02	.994	1.000
Global Severity Index (GSI)	0.88 (0.7)	0.90 (0.6)	0.87 (0.6)	0.06	2	0.05	.938	1.000
Gr. 2 (* n* = 29–30)								
F-INK								
Scale ‘participation'	1.31 (0.3)	1.26 (0.3)	1.34 (0.3)	1.18	2	0.20	.314	.627
Scale ‘inclusion'	1.98 (0.6)	1.92 (0.6)	1.95 (0.6)	0.51	1.7	0.14	.573	.627
BSI-18								
Scale ‘somatization'	1.02 (0.8)	0.90 (0.9)	0.76 (0.7)	1.50	2	0.23	.232	.232
Scale ‘depression'	1.62^a^ (1.2)	1.05^b^ (0.9)	1.01^b^ (0.9)	8.59	2	0.54	.001	.002
Scale ‘anxiety'	1.36^a^ (0.9)	1.03^b^ (0.8)	0.91^b^ (0.8)	6.15	1.6	0.46	.007	.015
Global Severity Index (GSI)	1.34^a^ (0.8)	0.99^b^ (0.8)	0.89^b^ (0.7)	6.53	1.7	0.48	.005	.014
Gr. 3 (* n* = 49)								
F-INK								
Scale ‘participation'	1.43^a^ (0.3)	1.25^b^ (0.2)	1.45^a^ (0.3)	21.07	1.8	0.66	< .001	< .001
Scale ‘inclusion'	2.44 (0.4)	2.40 (0.4)	2.38 (0.4)	1.27	2	0.16	.285	.286
BSI-18								
Scale ‘somatization'	0.25 (0.4)	0.22 (0.3)	0.22 (0.3)	0.38	2	0.09	.685	1.000
Scale ‘depression'	0.29 (0.5)	0.41 (0.5)	0.33 (0.5)	1.91	2	0.20	.154	.616
Scale ‘anxiety'	0.30 (0.4)	0.27 (0.3)	0.27 (0.3)	0.39	2	0.09	.676	1.000
Global Severity Index (GSI)	0.28 (0.3)	0.30 (0.3)	0.27 (0.3)	0.49	2	0.10	.612	1.000

.10 ≤ *f* < .25 = small effect, .25 ≤ *f* < .40 = medium effect, *f* ≥ .40 = large effect

^1^Possible range 0–4

^2^Possible range 0–3

Across all three groups and survey dates, feelings of inclusion, measured by the “inclusion module” of the F-INK, remained at a constant level (Table 4).

### Mental health (BSI-18)

As shown in Table 4, participants in Groups 1 and 3 reported unchanged mental health states in the week before the first follow-up survey and in the week before the second follow-up survey. In contrast, participants who were receiving acute psychiatric or psychotherapeutic treatment at the time of the initial survey (Group 2) indicated significantly improved scores on the subscales “depression” and “anxiety” of the BSI-18 as well as the Global Severity Index in the week before the first follow-up survey. These effects persisted through to the week before the second follow-up survey (Table 4). Detailed data showing the individual items of the BSI-18 can be found in Supplementary Tables 4.2.1 to 4.2.3.

## Discussion

In the present study, a variety of clinical and social psychiatric variables were investigated before, during and after COVID-19-related lockdown restrictions in three different groups: participants with a chronic mental disorder, participants who were receiving acute psychiatric or psychotherapeutic treatment at the beginning of the study, and participants from the general public who presented with neither acute nor chronic mental disorders. To our knowledge, this is the first time these variables have been so thoroughly investigated in relation to the COVID-19 pandemic.

The limitations of the present study include the relatively small number of participants in each of the three groups, the variable time periods between the pre- and post-surveys, as well as the regional specificity of the study. The results should therefore be interpreted with some care. Despite the small sample, it should be emphasised that non-significant results in the sum scores represented small or at most medium effects. However, a few large effects showed to be non-significant at the level of the single-item analyses, presumably due to the adjustment of the α-error. In addition, all our interpretations are based on mean values. As a result, there may of course have been stronger or weaker impairments in individual cases. Beyond this, only self-assessments and no external assessments were utilised to obtain data for the study. Lastly, generalisability of our findings is limited due to the sampling strategies and the baseline socio-demographic differences between the groups which may have contributed to the divergent effects of the pandemic.

However, the unique strength of the present study is that participants from multiple specific groups were examined longitudinally in an initial as well as two follow-up surveys. Using the data of the initial survey before the outbreak of the pandemic as a reference, we were able to measure the actual changes that have occurred over the course of time, i.e. when the lockdown restrictions and later the physical distancing policies were in force. Our results can be summarised as follows.

Participants with a chronic mental disorder showed no perceived impairment in their social participation as a result of the lockdown restrictions or the subsequent physical distancing policy associated with the COVID-19 pandemic. However, the impairments in the surveyed areas of everyday life were already at a relatively high level at the time of the initial survey for this group. This was further reflected in the minimal changes across the three surveys concerning the frequencies with which certain activities were carried out. These were, as could be expected [24, 25], already at a comparatively low level at the time of the initial survey, possibly indicating a floor effect. With regard to their mental health states, this group of participants showed high stability over time, indicating a high resilience to the official restrictions and measures as well as the pandemic itself.

Participants who were receiving acute psychiatric or psychotherapeutic treatment at the time of the initial survey reported significantly less impairments in several of the surveyed areas of everyday life during the lockdown restrictions and subsequent physical distancing restrictions, as compared to the time of the initial survey. The strong impairments at the time of the initial survey due to the acute mental disorder thus improved despite the adverse conditions resulting from the COVID-19 pandemic. This was to be expected given the participants’ likely recovery from their mental health condition during this time. While the frequencies with which certain activities were carried out changed only minimally in this group of participants, their mental health states improved significantly from the initial survey to the first follow-up survey and remained stable until the second follow-up survey. Overall, this suggests that resilience to the lockdown restrictions and the subsequent physical distancing policy as well as the pandemic as itself can also be assumed for this group.

Participants who presented with neither acute nor chronic mental disorders at the beginning of the study experienced extensive impairments in their social participation due to the lockdown restrictions. However, some of those areas of everyday life were only temporarily impaired and returned to normal levels by the time of the second follow-up survey when the restrictions had been largely lifted. The same applied to the extensive changes in behaviour of the participants associated with the lockdown restrictions. While a large proportion of activities were carried out less frequently during this time, most of them returned to pre-lockdown levels in the four weeks before to the second follow-up survey. Despite these temporary impairments and behavioural changes, the participants in this group did not report any negative effects on their mental health states, which again speaks for a resilience to the COVID-19 pandemic and the associated restrictions.

By completing a second follow-up survey, we have extended the findings of our previous study [8] and thus provided further evidence for a predominant resilience to the COVID-19 pandemic as well as the subsequent lockdown restrictions and physical distancing policy among the examined population groups. Although the lockdown restrictions may have led to more or less severe impairments in social participation and behavioural changes in our sample, these appeared to be tied to the strength of the official measures and, therefore, were only temporary. The feeling of inclusion, or more precisely the feeling of being structurally involved in and subjectively belonging to society, remained stable across all three groups and throughout the observation period. Interestingly, the restrictions also had no noticeable effect on the mental health states of the participants, whether or not they previously had a mental disorder.

Our results of resilience are therefore in concordance with other studies from Germany [6, 7] and New Zealand [5], but in discordance with findings from the UK [3], Italy [1], China [2] and Spain [4]. One reason for this could be that the population of Germany, and especially Dresden, has so far been less affected by the pandemic than other countries, e.g. in terms of the number of cases or the severity and duration of the restrictions. In addition, there have been no signs of an imminent overburdening of the German health care system and the confidence in the German government and key institutions in connection with the current pandemic seems to have remained at a medium-to-high level [26]. However, more studies are needed to further examine these assumptions. We, therefore, advocate that in future studies, a more detailed description of the pandemic situation in the respective study region should be provided. Information on the number of cases and local regulations at the time of the survey would help to improve the comparability of study results on this topic.

Finally, it should be highlighted that our results are in line with the observations of Martinelli and Ruggeri [12], who found an unexpectedly high degree of resilience during the pandemic lockdown in severely mentally ill users of supported accommodation services. Our study further shows, however, that social participation opportunities for individuals with chronic mental disorders are strongly limited, regardless of lockdown restrictions or physical distancing policies. Further efforts to improve social participation and inclusion opportunities are urgently needed to rectify this.

## Supplementary Information

Below is the link to the electronic supplementary material.

Supplementary material 1 (DOCX 68 kb)

## Data Availability

The datasets analysed during the current study and the F-INK questionnaire are available from the corresponding author upon reasonable request.

## References

[1] Pancani L, Marinucci M, Aureli N (2020). Forced social isolation and mental health: a study on 1006 Italians under COVID-19 lockdown. PsyArXiv.

[2] Zhang SX, Wang Y, Rauch A (2020). Unprecedented disruption of lives and work: Health, distress and life satisfaction of working adults in China one month into the COVID-19 outbreak. Psychiatry Res.

[3] Daly M, Sutin A, Robinson E (2020) Longitudinal changes in mental health and the COVID-19 pandemic: evidence from the UK Household Longitudinal Study. Psychol Med 1–10. 10.1017/S0033291720004432 10.1017/S0033291720004432 PMC7737138 33183370

[4] Gonzalez-Sanguino C, Ausín B, Castellanos MA (2020). Mental health consequences during the initial stage of the 2020 coronavirus pandemic (COVID-19) in Spain. Brain Behav Immun.

[5] Sibley CG, Greaves LM, Satherley N (2020). Effects of the COVID-19 pandemic and nationwide lockdown on trust, attitudes toward government, and well-being. Am Psychol.

[6] Entringer T, Kröger H (2020) Einsam, aber resilient—Die Menschen haben den Lockdown besser verkraftet als vermutet. [Lonesome but resilient—people coped with the lockdown better than expected]. DIW aktuell 46. DIW aktuell 46. https://doi.org/10419/222876

[7] Röhr S, Reininghaus U, Riedel-Heller S (2020) Mental and social health in the German old age population largely unaltered during COVID-19 lockdown: results of a representative survey. 10.31234/osf.io/7n2bm 10.1186/s12877-020-01889-x PMC7681185 33225912

[8] Schützwohl M, Mergel E (2020) Teilhabemöglichkeit, Partizipation, Inklusion und psychisches Befinden im Zusammenhang mit Ausgangsbeschränkungen aufgrund SARS-CoV-2: Ergebnisse einer Follow-up-Studie. [Social participation, inclusion and mental well-being following SARS-CoV-2 related restrictions on going out: a follow-up study from Germany]. Psychiat Prax. 10.1055/a-1202-2427 10.1055/a-1202-2427 32688427

[9] Vindegaard N, Benros ME (2020). COVID-19 pandemic and mental health consequences: systematic review of the current evidence. Brain Behav Immun.

[10] Hao F, Tan W, Jiang L (2020). Do psychiatric patients experience more psychiatric symptoms during COVID-19 pandemic and lockdown? A case-control study with service and research implications for immunopsychiatry. Brain Behav Immun.

[11] Gobbi S, Plomecka MB, Ashraf Z et al (2020) Worsening of pre-existing psychiatric conditions during the COVID-19 pandemic. 10.1101/2020.05.28.20116178

[12] Martinelli A, Ruggeri M (2020). The impact of COVID-19 on patients of Italian mental health supported accommodation services. Soc Psychiatry Psychiatr Epidemiol.

[13] Mækelæ MJ, Reggev N, Dutra N (2020). Perceived efficacy of COVID-19 restrictions, reactions and their impact on mental health during the early phase of the outbreak in six countries. R Soc Open Sci.

[14] Bäuerle A, Teufel M, Musche V (2020). Increased generalized anxiety, depression and distress during the COVID-19 pandemic: a cross-sectional study in Germany. J Public Health (Oxf).

[15] Qiu J, Shen B, Zhao M (2020). A nationwide survey of psychological distress among Chinese people in the COVID-19 epidemic: implications and policy recommendations. Gen Psychiatry.

[16] Schützwohl M, de Souza P, Rackel Y (2017). Fragebogen zur Erfassung von Partizipation und Inklusion chronisch psychisch erkrankter Menschen (F-INK). Entwicklung und Untersuchung teststatistischer Gütekriterien. [A measure of participation and social inclusion for use in people with a chronic mental disorder (F-INK)]. Psychiat Prax.

[17] Deck R, Mittag O, Hüppe A et al (2007) Index zur Messung von Einschränkungen der Teilhabe (IMET)—Erste Ergebnisse eines ICF-orientierten Assessmentinstrumentes. [Index for the Assessment of Health Impairments (IMET)—first findings from an ICF-oriented assessment instrument]. Klinische Verhaltensmedizin und Rehabilitation 76:113–120. 10.23668/psycharchives.381

[18] Spitzer C, Hammer S, Löwe B (2011). Die Kurzform des Brief Symptom Inventory (BSI-18): erste Befunde zu den psychometrischen Kennwerten der deutschen Version. [The short version of the Brief-Symptom Inventory (BSI-18): preliminary psychometric properties of the German translation]. Fortschr Neurol Psychiat.

[19] WHO Regional Office for Europe (2020) COVID-19 Snapshot Monitoring (COSMO Standard): monitoring knowledge, risk perceptions, preventive behaviours, and public trust in the current coronavirus outbreak—WHO standard protocol. 10.23668/psycharchives.2782

[20] Stadt Dresden (2020) Coronavirus. https://www.dresden.de/de/leben/gesundheit/hygiene/infektionsschutz/corona.php. Accessed 8 Sept 2020

[21] Sächsisches Staatsministerium für Soziales und Gesellschaftlichen Zusammenhalt (2020a) Allgemeinverfügung vom 22. März 2020. [Decree of the Saxon Ministry for Social Affairs and Social Cohesion for protection against coronavirus SARS-CoV-2 and COVID19 from March 22, 2020]. https://www.coronavirus.sachsen.de/download/AllgV-Corona-Ausgangsbeschraenkungen_22032020.pdf. Accessed 8 Sept 2020

[22] Sächsisches Staatsministerium für Soziales und Gesellschaftlichen Zusammenhalt (2020b) Allgemeinverfügung vom 17. April 2020. [Decree of the Saxon Ministry for Social Affairs and Social Cohesion for protection against coronavirus SARS-CoV-2 and COVID19 from April 17, 2020]. https://www.coronavirus.sachsen.de/download/SMS-SaechsCoronaSchVO-2020-04-17.pdf. Accessed 8 Sept 2020

[23] Sächsisches Staatsministerium für Soziales und Gesellschaftlichen Zusammenhalt (2020c) Allgemeinverfügung vom 25. Juni 2020. [Decree of the Saxon Ministry for Social Affairs and Social Cohesion for protection against coronavirus SARS-CoV-2 und COVID19 from June 25, 2020]. https://www.coronavirus.sachsen.de/download/SMS-Corona-Schutz-Verordnung-2020-06-25.pdf. Accessed 8 Sept 2020

[24] Boardman J (2011). Social exclusion and mental health–how people with mental health problems are disadvantaged: an overview. Ment Health Soc Inclus.

[25] Daum M, Höptner A, Speck A (2017). Teilhabe für chronisch psychisch kranke Menschen in Deutschland oder Die Sozialpsychiatrie und die Soziale Gerechtigkeit. Participation for chronically mentally ill people in Germany or Social psychiatry and social justice. Psychiatr Prax.

[26] Betsch C, Korn L, Felgendreff L et al (2020) German COVID-19 Snapshot Monitoring (COSMO)—Welle 17 (21.07.2020). [German COVID-19 Snapshot Monitoring (COSMO)—Wave 17 (21/07/2020)]. 10.23668/psycharchives.3156

