# Supplementary material for: A longitudinal study on the COVID-19 pandemic and its divergent effects on social participation and mental health across different study groups with and without mental disorders
Source: Soc Psychiatry Psychiatr Epidemiol. 2021 Feb 10;56(8):1459–68. doi: 10.1007/s00127-021-02025-9 (PMC7875168; doi:10.1007/s00127-021-02025-9)
Supplement: Supplementary file 1 — Supplementary material 1 (DOCX 68 kb) [file 127_2021_2025_MOESM1_ESM.docx]

**Supplementary Table 4.1.1. Participation (F-INK)**

|  | Gr. 1  (n=23-27) | | |  | | | | |
| --- | --- | --- | --- | --- | --- | --- | --- | --- |
|  | Initial survey | First follow-up survey (March 23 – April 20) | Second follow-up survey (June 22 – July 19) |  | | | | |
| Specific activities^1^ | *M* (*SD*) | *M* (*SD*) | *M* (*SD*) | *F* | *df* | *f* | *p* | *p_adj_* |
| *Buying groceries* | 2.52 (1.3) | 2.04 (1.2) | 2.22 (1.1) | 2.70 | 2 | .35 | .078 | 1.000 |
| *Visiting family members or friends* | 0.79 (0.7) | 0.75 (1.2) | 0.96 (1.0) | 0.42 | 1.4 | .14 | .586 | 1.000 |
| *Talking on the phone* | 2.48 (1.4) | 2.64 (1.2) | 2.64 (1.4) | 0.25 | 2 | .10 | .776 | 1.000 |
| *Paying bills/ withdrawing money* | 1.35 (1.0) | 1.00 (0.9) | 1.19 (1.2) | 1.05 | 2 | .20 | .356 | 1.000 |
| *Joining a party* | 0.70^a^ (0.8) | 0.04^b^ (0.2) | 0.22^b^ (0.7) | 8.19 | 2 | .61 | .001 | .030 |
| *Answering letters/ going to the post office* | 0.60 (0.9) | 0.56 (0.8) | 0.56 (0.8) | 0.05 | 1.5 | .04 | .916 | 1.000 |
| *Reading* | 2.15 (1.7) | 1.88 (1.6) | 1.46 (1.7) | 3.28 | 1.5 | .36 | .062 | 1.000 |
| *Going to a museum/ exhibition* | 0.22 (0.4) | 0.00 (0.0) | 0.00 (0.0) | 6.11 | 2 | .53 | .005 | .141 |
| *Cooking* | 2.56 (1.3) | 2.28 (1.4) | 2.52 (1.2) | 0.82 | 2 | .18 | .446 | 1.000 |
| *Going to a sports event* | 0.12 (0.6) | 0.00 (0.0) | 0.00 (0.0) | 1.00 | 2 | .20 | .375 | 1.000 |
| *Dealing with local authorities* | 0.32 (0.6) | 0.40 (0.6) | 0.32 (0.7) | 0.19 | 2 | .09 | .825 | 1.000 |
| *Going for a walk* | 1.96 (1.7) | 2.25 (1.6) | 2.00 (1.6) | 0.80 | 2 | .19 | .453 | 1.000 |
| *Having a day trip* | 0.39 (0.6) | 0.22 (0.7) | 0.17 (0.5) | 0.91 | 1.5 | .20 | .388 | 1.000 |
| *Joining a course* | 0.46 (0.9) | 0.25 (0.8) | 0.29 (0.8) | 0.46 | 2 | .14 | .637 | 1.000 |
| *Attending a vocational development* | 0.12 (0.4) | 0.08 (0.4) | 0.12 (0.6) | 0.05 | 2 | .04 | .948 | 1.000 |
| *Going for a swim* | 0.08 (0.3) | 0.00 (0.0) | 0.28 (0.7) | 3.59 | 1.3 | .39 | .058 | 1.000 |
| *Listening to music* | 2.76 (1.7) | 2.96 (1.5) | 2.68 (1.6) | 0.66 | 1.6 | .17 | .491 | 1.000 |
| *Going to a library* | 0.33 (0.9) | 0.08 (0.4) | 0.33 (0.9) | 1.41 | 2 | .25 | .255 | 1.000 |
| *Using messengers (e.g. WhatsApp)* | 2.21 (1.9) | 2.00 (1.8) | 1.83 (1.8) | 1.35 | 1.6 | .24 | .269 | 1.000 |
| *Playing a board game* | 0.92 (1.1) | 0.64 (1.2) | 0.84 (1.2) | 0.64 | 2 | .16 | .531 | 1.000 |
| *Meeting with friends* | 1.38 (1.5) | 0.83 (1.3) | 1.08 (1.4) | 2.31 | 2 | .32 | .111 | 1.000 |
| *Doing a sports workout* | 0.80 (1.3) | 0.60 (1.2) | 0.68 (1.3) | 0.33 | 2 | .12 | .720 | 1.000 |
| *Taking care of a pet* | 0.57 (1.4) | 0.52 (1.4) | 0.57 (1.4) | 1.00 | 2 | .21 | .376 | 1.000 |
| *Going to a bar/ restaurant* | 0.40 (0.6) | 0.12 (0.4) | 0.36 (0.8) | 2.26 | 2 | .31 | .116 | 1.000 |
| *Shopping in the internet* | 0.46 (0.8) | 0.46 (0.8) | 0.42 (0.9) | 0.09 | 1.6 | .05 | .878 | 1.000 |
| *Taking care of sb./ providing assistance* | 0.80 (1.2) | 0.56 (0.8) | 0.84 (1.2) | 0.98 | 2 | .20 | .384 | 1.000 |
| *Buying clothes* | 0.29 (0.6) | 0.25 (0.6) | 0.38 (0.5) | 0.43 | 2 | .14 | .655 | 1.000 |
| *Inviting guests* | 0.39 (0.9) | 0.17 (0.7) | 0.43 (0.9) | 1.11 | 1.4 | .22 | .322 | 1.000 |
| *Writing/ answering e-mails* | 1.00 (1.4) | 1.00 (1.4) | 1.08 (1.5) | 0.18 | 2 | .08 | .832 | 1.000 |
| *Joining a religious event* | 0.43 (0.8) | 0.09 (0.4) | 0.17 (0.6) | 3.62 | 1.5 | .41 | .048 | 1.000 |
| *Watching TV* | 3.27 (1.4) | 3.31 (1.3) | 3.23 (1.3) | 0.17 | 2 | .08 | .843 | 1.000 |
| *Going to a concert/ cinema/ theatre/ opera* | 0.16 (0.4) | 0.00 (0.0) | 0.04 (0.2) | 2.79 | 1.4 | .34 | .094 | 1.000 |

*Note*. In case the sphericity assumption was violated, a Huynh-Feldt correction was applied. The Bonferroni-Holm method was applied to obtain the adjusted p-values. Per row, means followed by a different letter significantly differ at 5% level according to post hoc tests.

.10 ≤ *f* < .25 = small effect, .25 ≤ *f* < .40 = medium effect, *f* ≥ .40 = large effect

^1^ [0]=never in the last four weeks – [4]=almost every day

**Supplementary Table 4.1.2. Participation (F-INK)**

|  | Gr. 2  (n=27-30) | | |  | | | | |
| --- | --- | --- | --- | --- | --- | --- | --- | --- |
|  | Initial survey | First follow-up survey (March 23 – April 20) | Second follow-up survey (June 22 – July 19) |  | | | | |
| Specific activities^1^ | *M* (*SD*) | *M* (*SD*) | *M* (*SD*) | *F* | *df* | *f* | *p* | *p_adj_* |
| *Buying groceries* | 2.73 (0.9) | 2.53 (0.9) | 2.77 (1.1) | 1.41 | 2 | .22 | .253 | 1.000 |
| *Visiting family members or friends* | 1.46 (1.3) | 1.14 (1.2) | 1.82 (1.3) | 6.32 | 2 | .48 | .003 | .102 |
| *Talking on the phone* | 2.93 (1.2) | 3.31 (0.7) | 3.28 (0.8) | 2.43 | 1.6 | .29 | .111 | 1.000 |
| *Paying bills/ withdrawing money* | 1.93 (0.9) | 1.72 (0.8) | 1.97 (0.6) | 1.11 | 1.7 | .20 | .331 | 1.000 |
| *Joining a party* | 0.52 (0.6) | 0.22 (0.5) | 0.59 (0.7) | 5.09 | 2 | .44 | .010 | .268 |
| *Answering letters/ going to the post office* | 0.97 (0.9) | 1.17 (0.9) | 1.20 (1.2) | 0.65 | 2 | .15 | .524 | 1.000 |
| *Reading* | 2.90 (1.3) | 3.03 (1.2) | 2.79 (1.4) | 0.44 | 2 | .12 | .648 | 1.000 |
| *Going to a museum/ exhibition* | 0.21 (0.6) | 0.07 (0.3) | 0.18 (0.4) | 0.86 | 1.6 | .18 | .410 | 1.000 |
| *Cooking* | 2.60 (1.2) | 2.87 (1.2) | 2.60 (1.2) | 0.82 | 2 | .17 | .447 | 1.000 |
| *Going to a sports event* | 0.43 (0.8) | 0.20 (0.6) | 0.20 (0.7) | 1.80 | 2 | .25 | .175 | 1.000 |
| *Dealing with local authorities* | 0.67 (0.8) | 0.33 (0.7) | 0.40 (0.7) | 2.31 | 2 | .28 | .109 | 1.000 |
| *Going for a walk* | 2.31 (1.3) | 2.86 (1.1) | 2.21 (1.2) | 6.02 | 2 | .46 | .004 | .124 |
| *Having a day trip* | 0.80 (1.0) | 0.53 (0.9) | 0.83 (0.7) | 1.27 | 1.7 | .21 | .286 | 1.000 |
| *Joining a course* | 0.40 (0.9) | 0.00 (0.0) | 0.33 (0.8) | 4.30 | 2 | .38 | .018 | .471 |
| *Attending a vocational development* | 0.10 (0.5) | 0.00 (0.0) | 0.03 (0.2) | 1.00 | 2 | .18 | .374 | 1.000 |
| *Going for a swim* | 0.27 (0.6) | 0.10 (0.5) | 0.40 (0.8) | 2.04 | 2 | .27 | .140 | 1.000 |
| *Listening to music* | 2.93 (1.5) | 2.89 (1.5) | 3.04 (1.3) | 0.19 | 2 | .08 | .826 | 1.000 |
| *Going to a library* | 0.14 (0.4) | 0.07 (0.4) | 0.17 (0.5) | 0.69 | 1.7 | .16 | .479 | 1.000 |
| *Using messengers (e.g. WhatsApp)* | 3.41 (1.2) | 3.41 (1.3) | 3.45 (1.3) | 0.14 | 1.7 | .07 | .833 | 1.000 |
| *Playing a board game* | 0.77 (0.9) | 0.93 (1.0) | 0.87 (1.0) | 0.42 | 2 | .12 | .657 | 1.000 |
| *Meeting with friends* | 1.45 (1.3) | 0.97 (1.3) | 1.55 (1.5) | 3.59 | 2 | .36 | .034 | .851 |
| *Doing a sports workout* | 1.45 (1.4) | 1.79 (1.5) | 1.69 (1.4) | 1.09 | 2 | .20 | .345 | 1.000 |
| *Taking care of a pet* | 1.17 (1.8) | 0.90 (1.6) | 0.72 (1.5) | 2.02 | 1.6 | .27 | .152 | 1.000 |
| *Going to a bar/ restaurant* | 0.60^a^ (0.7) | 0.03^b^ (0.2) | 0.80^a^ (1.0) | 13.87 | 2 | .69 | <.001 | <.001 |
| *Shopping in the internet* | 0.77 (0.9) | 1.17 (1.1) | 0.90 (0.9) | 4.46 | 2 | .39 | .016 | .426 |
| *Taking care of sb./ providing assistance* | 1.48 (1.7) | 1.48 (1.5) | 1.11 (1.3) | 1.04 | 2 | .20 | .360 | 1.000 |
| *Buying clothes* | 0.62 (0.7) | 0.38 (0.6) | 0.52 (0.6) | 1.81 | 2 | .25 | .173 | 1.000 |
| *Inviting guests* | 0.59 (1.0) | 0.33 (0.7) | 0.70 (1.0) | 3.61 | 2 | .37 | .034 | .851 |
| *Writing/ answering e-mails* | 2.44 (1.2) | 2.48 (1.2) | 2.44 (1.1) | 0.02 | 2 | .03 | .984 | 1.000 |
| *Joining a religious event* | 0.03 (0.2) | 0.07 (0.3) | 0.10 (0.3) | 0.59 | 2 | .15 | .557 | 1.000 |
| *Watching TV* | 3.00 (1.4) | 3.17 (1.5) | 2.97 (1.6) | 1.50 | 2 | .23 | .232 | 1.000 |
| *Going to a concert/ cinema/ theatre/ opera* | 0.52^a^ (0.6) | 0.03^b^ (0.2) | 0.24^c^ (0.4) | 10.28 | 1.6 | .61 | .001 | .017 |

*Note*. In case the sphericity assumption was violated, a Huynh-Feldt correction was applied. The Bonferroni-Holm method was applied to obtain the adjusted p-values. Per row, means followed by a different letter significantly differ at 5% level according to post hoc tests.

.10 ≤ *f* < .25 = small effect, .25 ≤ *f* < .40 = medium effect, *f* ≥ .40 = large effect

^1^ [0]=never in the last four weeks – [4]=almost every day

**Supplementary Table 4.1.3. Participation (F-INK)**

|  | Gr. 3  (n=46-49) | | |  | | | | |
| --- | --- | --- | --- | --- | --- | --- | --- | --- |
|  | Initial survey | First follow-up survey (March 23 – April 20) | Second follow-up survey (June 22 – July 19) |  | | | | |
| Specific activities^1^ | *M* (*SD*) | *M* (*SD*) | *M* (*SD*) | *F* | *df* | *f* | *p* | *p_adj_* |
| *Buying groceries* | 2.63^a^ (0.9) | 2.16^b^ (0.9) | 2.55^a^ (0.9) | 7.82 | 2 | .40 | .001 | .016 |
| *Visiting family members or friends* | 1.85^a^ (1.0) | 1.04^b^ (1.1) | 1.92^a^ (1.0) | 13.94 | 2 | .55 | <.001 | <.001 |
| *Talking on the phone* | 2.91 (1.0) | 3.07 (0.8) | 3.09 (0.9) | 0.95 | 2 | .15 | .391 | 1.000 |
| *Paying bills/ withdrawing money* | 1.66^a^ (0.8) | 1.30^b^ (0.8) | 1.70^a^ (0.8) | 6.53 | 2 | .38 | .002 | .043 |
| *Joining a party* | 0.87^a^ (0.8) | 0.02^b^ (0.1) | 0.65^a^ (0.6) | 33.19 | 1.8 | .84 | <.001 | <.001 |
| *Answering letters/ going to the post office* | 0.90 (1.0) | 1.29 (1.1) | 1.23 (1.1) | 4.61 | 2 | .31 | .012 | .173 |
| *Reading* | 3.02 (1.2) | 2.92 (1.4) | 2.94 (1.2) | 0.22 | 2 | .06 | .807 | 1.000 |
| *Going to a museum/ exhibition* | 0.38^a^ (0.6) | 0.00^b^ (0.0) | 0.29^a^ (0.7) | 9.93 | 2 | .46 | <.001 | .003 |
| *Cooking* | 2.87 (1.0) | 3.06 (1.2) | 3.17 (1.0) | 2.56 | 2 | .23 | .083 | .747 |
| *Going to a sports event* | 0.30 (0.7) | 0.02 (0.1) | 0.09 (0.3) | 6.17 | 1.2 | .37 | .012 | .173 |
| *Dealing with local authorities* | 0.27 (0.5) | 0.12 (0.4) | 0.27 (0.5) | 1.66 | 2 | .18 | .196 | 1.000 |
| *Going for a walk* | 2.16^a^ (1.1) | 3.08^b^ (1.0) | 2.47^c^ (1.2) | 22.41 | 2 | .68 | <.001 | <.001 |
| *Having a day trip* | 1.04^a^ (0.9) | 0.31^b^ (0.7) | 1.10^a^ (0.9) | 18.66 | 2 | .62 | <.001 | <.001 |
| *Joining a course* | 0.47^a^ (0.9) | 0.02^b^ (0.1) | 0.30^a^ (0.7) | 7.70 | 1.8 | .41 | .001 | .028 |
| *Attending a vocational development* | 0.33 (0.9) | 0.00 (0.0) | 0.21 (0.7) | 3.20 | 1.6 | .26 | .057 | .573 |
| *Going for a swim* | 0.28^a^ (0.6) | 0.00^b^ (0.0) | 0.49^a^ (0.8) | 9.41 | 1.7 | .45 | <.001 | .009 |
| *Listening to music* | 3.48 (0.8) | 3.44 (1.0) | 3.29 (0.9) | 1.66 | 2 | .19 | .196 | 1.000 |
| *Going to a library* | 0.47^a^ (1.0) | 0.02^b^ (0.1) | 0.32^a^ (0.7) | 7.19 | 1.8 | .40 | .002 | .040 |
| *Using messengers (e.g. WhatsApp)* | 3.73 (0.9) | 3.88 (0.6) | 3.86 (0.6) | 2.79 | 1.3 | .24 | .089 | .747 |
| *Playing a board game* | 0.62 (0.8) | 1.08 (1.2) | 0.98 (1.0) | 5.53 | 2 | .34 | .005 | .086 |
| *Meeting with friends* | 1.91^a^ (1.0) | 0.64^b^ (0.9) | 1.77^a^ (0.9) | 43.71 | 2 | .97 | <.001 | <.001 |
| *Doing a sports workout* | 1.40 (1.3) | 1.94 (1.4) | 1.85 (1.2) | 4.69 | 1.8 | .32 | .015 | .177 |
| *Taking care of a pet* | 1.10 (1.7) | 1.13 (1.7) | 1.40 (1.8) | 2.03 | 2 | .21 | .137 | .958 |
| *Going to a bar/ restaurant* | 1.13^a^ (0.8) | 0.00^b^ (0.0) | 1.00^a^ (0.9) | 48.32 | 2 | 1.01 | <.001 | <.001 |
| *Shopping in the internet* | 1.20 (0.9) | 1.31 (1.1) | 1.29 (0.8) | 0.35 | 1.6 | .08 | .665 | 1.000 |
| *Taking care of sb./ providing assistance* | 1.24 (1.6) | 1.24 (1.5) | 1.20 (1.5) | 0.04 | 1.8 | .03 | .952 | 1.000 |
| *Buying clothes* | 0.50^a^ (0.6) | 0.29^b^ (0.5) | 0.65^a^ (0.6) | 6.41 | 2 | .37 | .002 | .044 |
| *Inviting guests* | 0.61^a^ (0.7) | 0.07^b^ (0.2) | 0.70^a^ (0.7) | 19.65 | 2 | .66 | <.001 | <.001 |
| *Writing/ answering e-mails* | 2.55 (1.4) | 2.92 (1.2) | 2.71 (1.1) | 3.57 | 1.7 | .27 | .039 | .425 |
| *Joining a religious event* | 0.20 (0.5) | 0.02 (0.1) | 0.02 (0.1) | 5.94 | 1.3 | .35 | 0.12 | .173 |
| *Watching TV* | 3.02 (1.2) | 3.31 (1.2) | 3.08 (1.4) | 5.78 | 2 | .35 | .004 | .073 |
| *Going to a concert/ cinema/ theatre/ opera* | 0.69^a^ (0.9) | 0.00^b^ (0.0) | 0.16^c^ (0.5) | 22.84 | 1.6 | .69 | <.001 | <.001 |

*Note*. In case the sphericity assumption was violated, a Huynh-Feldt correction was applied. The Bonferroni-Holm method was applied to obtain the adjusted p-values. Per row, means followed by a different letter significantly differ at 5% level according to post hoc tests.

.10 ≤ *f* < .25 = small effect, .25 ≤ *f* < .40 = medium effect, *f* ≥ .40 = large effect

^1^ [0]=never in the last four weeks – [4]=almost every day

**Supplementary Table 4.2.1. Mental health problems (BSI-18)**

|  | Gr. 1  (n=24-27) | | |  | | | | |
| --- | --- | --- | --- | --- | --- | --- | --- | --- |
|  | Initial survey | First follow-up survey (March 23 – April 20) | Second follow-up survey (June 22 – July 19) |  | | | | |
| Symptoms (last 7 days)^1^ | *M* (*SD*) | *M* (*SD*) | *M* (*SD*) | *F* | *df* | *f* | *p* | *p_adj_* |
| *Faintess or dizziness* | 0.56 (0.9) | 0.52 (0.7) | 0.72 (1.0) | 0.72 | 2 | .17 | .494 | 1.000 |
| *Pains in heart or chest* | 0.36 (0.6) | 0.52 (0.7) | 0.28 (0.7) | 1.29 | 2 | .23 | .285 | 1.000 |
| *Nausea or upset stomach* | 0.52 (0.8) | 0.40 (0.7) | 0.56 (1.1) | 0.56 | 2 | .15 | .578 | 1.000 |
| *Trouble getting your breath* | 0.40 (0.7) | 0.56 (0.9) | 0.60 (0.9) | 0.65 | 2 | .16 | .528 | 1.000 |
| *Numbness or tingling in parts of your body* | 0.56 (0.8) | 1.08 (1.2) | 1.12 (1.0) | 3.61 | 2 | .39 | .035 | .624 |
| *Feeling weak in parts of your body* | 1.16 (1.2) | 0.72 (0.8) | 1.00 (1.2) | 2.33 | 2 | .31 | .108 | 1.000 |
| *Feeling no interest in things* | 0.92 (1.2) | 0.88 (1.0) | 1.00 (1.2) | 0.22 | 2 | .10 | .805 | 1.000 |
| *Feeling lonely* | 1.24 (1.3) | 1.48 (1.4) | 1.16 (1.3) | 0.72 | 2 | .17 | .490 | 1.000 |
| *Feeling blue* | 0.92 (1.0) | 1.32 (1.4) | 1.04 (1.0) | 1.62 | 1.4 | .26 | .215 | 1.000 |
| *Feelings of worthlessness* | 1.29 (1.4) | 1.21 (1.3) | 1.08 (1.3) | 0.35 | 2 | .12 | .707 | 1.000 |
| *Feeling hopeless about the future* | 1.54 (1.2) | 1.38 (1.5) | 1.29 (1.3) | 0.71 | 2 | .18 | .497 | 1.000 |
| *Thoughts of ending your life* | 0.52 (1.0) | 0.40 (0.9) | 0.48 (1.0) | 0.57 | 2 | .15 | .567 | 1.000 |
| *Nervousness or shakiness inside* | 1.35 (1.2) | 1.27 (1.1) | 1.23 (1.2) | 0.17 | 2 | .08 | .845 | 1.000 |
| *Feeling tense or keyed up* | 1.72 (1.4) | 1.64 (1.4) | 1.56 (1.2) | 0.19 | 2 | .09 | .827 | 1.000 |
| *Suddenly scared for no reasons* | 0.24 (0.4) | 0.20 (0.5) | 0.28 (0.7) | 0.14 | 1.7 | .08 | .831 | 1.000 |
| *Spells of terror or panic* | 0.54 (1.1) | 0.65 (0.9) | 0.50 (0.8) | 0.40 | 2 | .13 | .675 | 1.000 |
| *Feeling so restless you couldn’t sit still* | 0.60 (1.0) | 0.76 (1.1) | 1.04 (1.1) | 2.11 | 2 | .30 | .132 | 1.000 |
| *Feeling fearful* | 0.92 (1.2) | 1.08 (1.2) | 0.88 (1.1) | 0.63 | 2 | .16 | .539 | 1.000 |

*Note*. In case the sphericity assumption was violated, a Huynh-Feldt correction was applied. The Bonferroni-Holm method was applied to obtain the adjusted p-values. Per row, means followed by a different letter significantly differ at 5% level according to post hoc tests.

.10 ≤ *f* < .25 = small effect, .25 ≤ *f* < .40 = medium effect, *f* ≥ .40 = large effect

^1^ [0]=not at all – [4]=extremely

**Supplementary Table 4.2.2. Mental health problems (BSI-18)**

|  | Gr. 2  (n=29-30) | | |  | | | | |
| --- | --- | --- | --- | --- | --- | --- | --- | --- |
|  | Initial survey | First follow-up survey (March 23 – April 20) | Second follow-up survey (June 22 – July 19) |  | | | | |
| Symptoms (last 7 days)^1^ | *M* (*SD*) | *M* (*SD*) | *M* (*SD*) | *F* | *df* | *f* | *p* | *p_adj_* |
| *Faintess or dizziness* | 1.28 (1.1) | 0.66 (0.9) | 0.76 (0.8) | 4.86 | 2 | .42 | .011 | .147 |
| *Pains in heart or chest* | 0.69 (1.0) | 0.93 (1.2) | 0.79 (1.1) | 0.82 | 2 | .17 | .447 | 1.000 |
| *Nausea or upset stomach* | 1.28 (1.2) | 1.00 (1.3) | 0.97 (1.2) | 0.78 | 2 | .17 | .464 | 1.000 |
| *Trouble getting your breath* | 0.93 (1.3) | 1.03 (1.2) | 0.63 (1.1) | 1.95 | 2 | .26 | .152 | 1.000 |
| *Numbness or tingling in parts of your body* | 0.67 (1.0) | 0.73 (1.1) | 0.67 (0.8) | 0.07 | 2 | .04 | .934 | 1.000 |
| *Feeling weak in parts of your body* | 1.28 (1.3) | 1.00 (1.1) | 0.79 (1.0) | 2.92 | 1.8 | .32 | .070 | .628 |
| *Feeling no interest in things* | 1.66 (1.5) | 1.07 (1.3) | 1.10 (1.1) | 3.49 | 2 | .35 | .037 | .374 |
| *Feeling lonely* | 1.87 (1.3) | 1.33 (1.1) | 1.27 (1.1) | 4.02 | 2 | .37 | .023 | .256 |
| *Feeling blue* | 1.83^a^ (1.4) | 0.97^b^ (1.1) | 1.14^b^ (1.4) | 11.04 | 2 | .63 | <.001 | .002 |
| *Feelings of worthlessness* | 1.50 (1.5) | 1.20 (1.3) | 1.03 (1.4) | 2.12 | 2 | .27 | .130 | 1.000 |
| *Feeling hopeless about the future* | 1.87 (1.5) | 1.30 (1.2) | 1.17 (1.3) | 5.50 | 2 | .43 | .007 | .098 |
| *Thoughts of ending your life* | 1.03 (1.5) | 0.50 (1.0) | 0.40 (0.9) | 5.17 | 1.7 | .42 | .012 | .147 |
| *Nervousness or shakiness inside* | 1.93^a^ (1.2) | 1.20^b^ (1.2) | 1.17^b^ (1.1) | 7.65 | 2 | .51 | .001 | .019 |
| *Feeling tense or keyed up* | 2.41 (1.1) | 2.03 (1.0) | 1.72 (1.1) | 5.86 | 1.6 | .46 | .009 | .131 |
| *Suddenly scared for no reasons* | 0.72 (1.2) | 0.62 (0.9) | 0.52 (0.8) | 0.81 | 2 | .17 | .449 | 1.000 |
| *Spells of terror or panic* | 0.63 (1.1) | 0.60 (1.1) | 0.57 (0.9) | 0.06 | 1.7 | .04 | .918 | 1.000 |
| *Feeling so restless you couldn’t sit still* | 1.07 (1.2) | 0.73 (0.9) | 0.77 (1.0) | 1.49 | 2 | .23 | .233 | 1.000 |
| *Feeling fearful* | 1.37 (1.3) | 0.97 (1.1) | 0.67 (1.1) | 6.32 | 1.7 | .47 | .006 | .093 |

*Note*. In case the sphericity assumption was violated, a Huynh-Feldt correction was applied. The Bonferroni-Holm method was applied to obtain the adjusted p-values. Per row, means followed by a different letter significantly differ at 5% level according to post hoc tests.

.10 ≤ *f* < .25 = small effect, .25 ≤ *f* < .40 = medium effect, *f* ≥ .40 = large effect

^1^ [0]=not at all – [4]=extremely

**Supplementary Table 4.2.3. Mental health problems (BSI-18)**

|  | Gr. 3  (n=48-49) | | |  | | | | |
| --- | --- | --- | --- | --- | --- | --- | --- | --- |
|  | Initial survey | First follow-up survey (March 23 – April 20) | Second follow-up survey (June 22 – July 19) |  | | | | |
| Symptoms (last 7 days)^1^ | *M* (*SD*) | *M* (*SD*) | *M* (*SD*) | *F* | *df* | *f* | *p* | *p_adj_* |
| *Faintess or dizziness* | 0.16 (0.4) | 0.24 (0.5) | 0.22 (0.6) | 0.51 | 2 | .11 | .599 | 1.000 |
| *Pains in heart or chest* | 0.18 (0.5) | 0.16 (0.4) | 0.16 (0.5) | 0.04 | 2 | .03 | .958 | 1.000 |
| *Nausea or upset stomach* | 0.33 (0.7) | 0.18 (0.4) | 0.16 (0.4) | 2.81 | 1.8 | .24 | .072 | 1.000 |
| *Trouble getting your breath* | 0.29 (0.8) | 0.24 (0.7) | 0.18 (0.6) | 1.27 | 1.6 | .16 | .280 | 1.000 |
| *Numbness or tingling in parts of your body* | 0.37 (0.7) | 0.29 (0.5) | 0.39 (0.8) | 0.52 | 2 | .11 | .596 | 1.000 |
| *Feeling weak in parts of your body* | 0.18 (0.4) | 0.22 (0.4) | 0.18 (0.4) | 0.25 | 2 | .07 | .782 | 1.000 |
| *Feeling no interest in things* | 0.31 (0.6) | 0.47 (0.8) | 0.41 (0.7) | 1.41 | 2 | .17 | .249 | 1.000 |
| *Feeling lonely* | 0.47 (0.6) | 0.76 (1.0) | 0.43 (0.7) | 3.53 | 2 | .27 | .033 | .599 |
| *Feeling blue* | 0.31 (0.7) | 0.44 (0.8) | 0.33 (0.7) | 0.79 | 2 | .13 | .456 | 1.000 |
| *Feelings of worthlessness* | 0.20 (0.5) | 0.10 (0.4) | 0.22 (0.4) | 2.27 | 2 | .22 | .109 | 1.000 |
| *Feeling hopeless about the future* | 0.42 (0.8) | 0.65 (0.9) | 0.50 (0.9) | 2.11 | 2 | .21 | .126 | 1.000 |
| *Thoughts of ending your life* | 0.04 (0.3) | 0.06 (0.3) | 0.08 (0.3) | 0.60 | 1.1 | .11 | .465 | 1.000 |
| *Nervousness or shakiness inside* | 0.35 (0.8) | 0.35 (0.7) | 0.24 (0.5) | 0.78 | 2 | .13 | .462 | 1.000 |
| *Feeling tense or keyed up* | 0.90 (0.9) | 0.73 (0.8) | 0.92 (0.8) | 1.00 | 2 | .14 | .372 | 1.000 |
| *Suddenly scared for no reasons* | 0.04 (0.2) | 0.04 (0.2) | 0.10 (0.4) | 1.83 | 1.6 | .20 | .174 | 1.000 |
| *Spells of terror or panic* | 0.10 (0.5) | 0.04 (0.2) | 0.02 (0.1) | 0.93 | 1.4 | .14 | .370 | 1.000 |
| *Feeling so restless you couldn’t sit still* | 0.14 (0.4) | 0.24 (0.4) | 0.18 (0.4) | 0.86 | 2 | .14 | .426 | 1.000 |
| *Feeling fearful* | 0.27 (0.6) | 0.18 (0.6) | 0.12 (0.4) | 2.38 | 1.8 | .22 | .103 | 1.000 |

*Note*. In case the sphericity assumption was violated, a Huynh-Feldt correction was applied. The Bonferroni-Holm method was applied to obtain the adjusted p-values. Per row, means followed by a different letter significantly differ at 5% level according to post hoc tests.

.10 ≤ *f* < .25 = small effect, .25 ≤ *f* < .40 = medium effect, *f* ≥ .40 = large effect

^1^ [0]=not at all – [4]=extremely
